# The Wounded Healer

**Published:** 2017-07-01

**Authors:** Morgan Gonzales, Laura Melton

**Affiliations:** Ms. Gonzales is a clinical social worker at the University of Colorado Hospital. Dr. Melton is the Medical Director of Supportive Oncology and an Assistant Professor in the Division of Medical Oncology at the University of Colorado.

Oncology clinicians skillfully and passionately provide services to patients with life-threatening cancer and are impacted daily by pain, loss, trauma, death, and grief. We understand that healing is not exclusively about treating or curing a disease, but also about finding meaning and peace while in the midst of pain and loss that cannot be changed. However, as we attend to the physical and psychosocial wounds our patients have incurred with a cancer diagnosis, we often try to hide our own wounds (D. Mattinson, personal communication, 2014). 

## MEMORABLE TRAUMATIC EXPERIENCE

As a new social worker in the Child Protective Services (CPS) field, I [MG] found myself in a constant state of survival following 4 years of consistent and relentless exposure to traumatic experiences. One of these instances was on a Saturday as I was on call for CPS. I was coaching youth soccer when I received an emergency call that I was needed at the hospital immediately. I arrived to find multiple police officers and detectives in the intensive care unit (ICU), as well as two frightened children wrapped in heating blankets. The children were extremely emaciated, and each had multiple physical wounds. (This was classified as the worst abuse case in the state at the time.) When interviewing these children, they repeatedly tried to convince me that they regularly ate large amounts of food and that the wounds on their bodies were from playing. Over the next 10 days of their ICU stay, I found myself in a tailspin. Monitoring the two sick children, attending multiple court hearings, and finding placement for the children in foster care took all of my physical and emotional energy. I continued to visit the children weekly while they were in foster care. 

Five weeks after meeting them for the first time under such dire conditions, the older child said to me, "Seeing you reminds me of what happened." I immediately disconnected. I was wounded, and the months I spent in employee health counseling and the extended amount of time I took off from work did not change that. I was wounded from the persistent impact of seeing sadness and trauma, being part of the ongoing trauma of these children, and being unable to process and label my feelings. 

## INTERPERSONAL PARADOX

Years later, I had moved into oncology, where I continued to struggle daily with relating to my patients. I saw multiple sad stories and difficult patients but never once felt the urge to reach out to comfort or allow sadness into my emotional realm. I distinctly remember sitting in a conference room, half listening to a webinar, and hearing the term *wounded healer* (Dickens & Partington, 2013). I had an immediate, visceral response to the phrase. Oncology clinicians are not wounded. I prided myself on being able to hold boundaries with patients and with myself. My response to this was, "Well, if you can’t handle it, then you should not do this work."

For weeks I continued to be haunted by this concept of the wounded healer, until one day, I had an "aha" moment. I was meeting with a young breast cancer patient in my normal guarded demeanor. She was mourning her loss of function and was starting to think about hospice. I sat on her bed, consciously put my hand on her shoulder (which was out of character for me), and allowed myself just to be with her. In that moment, I realized I too was wounded. 

Based on the Greek myth of the centaur Chiron and popularized by the psychiatrist Carl Jung, the *wounded healer* is a paradoxical concept (Groesbeck, 1975): The mythical healer heals all others but is never fully able to heal their own personal wound. This creates an interpersonal paradoxical dynamic where within the creation of wounds, healing can occur. As healers, we are expected to be whole and to find and fix wounds in our patients. However, if we can embrace our own *woundedness*, we can find relatedness. We avoid suffering only at the greatest cost of distancing ourselves from life. To live fully, we may need to look deeply and respectfully at our own suffering and at the suffering of others. 

In my deep-seated sadness, I found I was under the "M&M effect": I was hard on the outside but soft on the inside. Allowing the M&M effect to occur was impacting my practice with patients; I became hardened and unable to connect with them. I continued to identify myself as someone who had seen the worst and could deal with whatever came my way. I was proud of not being vulnerable and open to my patients. I had to realize this was not pride, but a wound so open and deep that I had just continued to put salt on it until it developed a hard crust.

I discovered that finding your wounds only comes at a time of deep reflection and meaning-making with yourself (Nouwen, 1979). I embraced my wounds and allowed healing to happen. I made meaning of the wound that I had avoided and detached from. I discovered a way not to allow it to stunt me, but instead to grow from it. I embraced, with some embarrassment, that I was OK to be affected by my patients. It was acceptable to be sad, to understand, and to let go and just be with a patient (Remen, 2000). 

## STRENGTH AND BEAUTY OF WOUNDS

We can benefit from exploring and acknowledging the strength and beauty of our wounds as we seek to heal others. In embracing our own *woundedness*, we can offer a deep presence that only comes from awareness that our wounds are what make us who we are and connect us to others (Remen, 2000). To identify our own wounds and become healed, we need to intentionally be present with ourselves, mindful. If we can have intention, purpose, and mindfulness in our time with ourselves and with our patients, we can heal from our own wounds. Healing our own wounds can decrease burnout and professional fatigue. We have to find a way to become whole through the sadness, trauma, and death we see daily (Remen, 2000). 

Working on owning our wounds can be stressful, tiring, and painful. We must remember that to do work on ourselves and our patients, we must become passionate advocates for ourselves (D. Mattinson, personal communication, 2015). Challenge yourself to have intentional self-care. Be present with yourself. Take time to breathe and reconnect with yourself, whether through an intentional mindfulness practice, seeking feedback with your peers, professional education, prayer, or exercise (Kabat-Zinn, 2012). Think about your challenges in life and work, become aware of how they impact you and your life at work with your patients, and draw yourself in.
